# The effect of pregnancy on the population pharmacokinetics of levofloxacin in South Africans with rifampicin-resistant tuberculosis

**DOI:** 10.1128/aac.01626-24

**Published:** 2025-04-01

**Authors:** Sharon Sawe, Lufina Tsirizani, Richard Court, Kamunkhwala Gausi, Asanda Poswa, Tasnim Badat, Lubbe Wiesner, Marian Loveday, Gary Maartens, Francesca Conradie, Paolo Denti

**Affiliations:** 1 Division of Clinical Pharmacology, Department of Medicine, University of Cape Town 71984, Cape Town, South Africa; 2 Department of Clinical Medicine, University of the Witwatersrand 56399, Johannesburg, South Africa; 3 HIV and Other Infectious Diseases Research Unit (HIDRU), South African Medical Research Council 59097, Durban, South Africa; St. George's, University of London, London, United Kingdom

**Keywords:** pregnancy, levofloxacin, NONMEM, serum creatinine, allometric scaling, dosing recommendations, population pharmacokinetic modelling, fluoroquinolones, renal function

## Abstract

Levofloxacin is a key drug in the prevention and treatment of rifampicin-resistant tuberculosis (RR-TB). There are limited data describing the effect of pregnancy on the pharmacokinetics of levofloxacin. We aimed to characterize the pharmacokinetics of levofloxacin in adults with RR-TB, including the effect of pregnancy. We pooled data from two studies conducted in adult participants treated for RR-TB in South Africa. Treatment regimens in both studies included levofloxacin dosed at 750/1000 mg daily, depending on body weight. We analyzed data from 47 participants, 31 (66%) living with HIV, using nonlinear mixed-effects modeling in NONMEM v7.5.1. Out of 33 female participants, 21 were pregnant, of whom 12 contributed matched antepartum and postpartum pharmacokinetic profiles. Levofloxacin followed one-compartment pharmacokinetics with first-order elimination and absorption with transit absorption compartments. The clearance and volume of distribution for a typical non-pregnant participant (weight: 58 kg; age: 32 years; serum creatinine: 56.2 µmol/L) were 6.06 (95% confidence interval [CI], 5.47 to 6.53) L/h and 85.9 (95% CI, 80.6 to 91.7) L, respectively. Higher serum creatinine levels were associated with lower levofloxacin clearance using a power function with an exponent of −0.367 (95% CI, −0.493 to −0.104). Pregnancy increased levofloxacin clearance by 38.1% (95% CI, 23.4% to 57.1%), with substantially lower exposures in pregnant compared with non-pregnant participants receiving equivalent weight-based doses. To achieve non-pregnant equivalent exposures of levofloxacin, an additional 250 mg tablet may be required, although further study is needed to assess the safety implications of a higher recommended dose in pregnant women.

## INTRODUCTION

Rifampicin-resistant tuberculosis (RR-TB), which may include resistance to other first-line anti-TB drugs (1), poses a significant challenge to the World Health Organization’s (WHO) 2030 End TB Strategy (2). Recent advances in RR-TB treatment have introduced shorter, all-oral regimens, such as the 2022 WHO-recommended 6-month BPAL/M regimen (bedaquiline, pretomanid, linezolid, +/-moxifloxacin), proven effective in previous trials (3). These regimens are also cost-effective (4) but face limitations, such as moxifloxacin’s higher risk of QTc prolongation, making levofloxacin preferable (5). Despite these advances, challenges remain, particularly for pregnant and postpartum women, who are at increased risk of active TB infection and RR-TB (6). Globally, an estimated 200,000 pregnant and postpartum women contract TB annually, and effective treatment of these patients while ensuring maternal and fetal safety is critical (7). Pregnant women have historically been excluded from clinical trials, so data on the safety and pharmacokinetics of RR-TB drugs in pregnancy are therefore limited (8).

Levofloxacin, a Group A fluoroquinolone antibiotic (3), is being used with increasing frequency for TB preventive therapy, with studies supporting safety in children and adults (9–11). Levofloxacin is rapidly absorbed, with bioavailability approaching 100%. The terminal half-life of levofloxacin in plasma ranges between 6 and 8 h in individuals with normal renal function (creatinine clearance >80 mL/min) (12). Levofloxacin is 24%–38% bound to plasma proteins (mainly serum albumin), undergoes minimal metabolism, and is primarily excreted as an unchanged drug in urine (12). The typical maximum concentration (C_max_) of levofloxacin at the current dose levels of tuberculosis ranges from 8 to 13 µg/mL (13). The ratio of the free area under the concentration–time curve over 24 h to the minimum inhibitory concentration (*f*AUC_0-24_/MIC) is used as the most relevant pharmacokinetic–pharmacodynamic (PK-PD) parameter for predicting optimal efficacy (14).

Physiological changes during pregnancy could influence the pharmacokinetics of levofloxacin through well-characterized mechanisms. Elevated gastric pH during pregnancy may impair the solubility and dissolution of levofloxacin, potentially reducing its absorption and bioavailability (15). Increased plasma volume and total body water may expand the volume of distribution, potentially resulting in lower peak plasma concentrations of the drug (16). Enhanced renal clearance during pregnancy, driven by a rise in glomerular filtration rate (GFR), reduces serum creatinine levels and facilitates the elimination of renally excreted drugs such as levofloxacin (17). Additionally, pregnancy-related increases in cardiac output enhance hepatic and renal blood flow, potentially influencing both renal and non-renal drug clearance (18). These physiological changes collectively suggest a potential reduction in levofloxacin exposure during pregnancy, which warrants further investigation to optimize dosing regimens in pregnant individuals.

We aimed to characterize the pharmacokinetics of levofloxacin in patients with RR-TB and evaluate the effect of pregnancy, body size, renal function, and HIV status on its exposure. Additionally, we used Monte Carlo simulations to explore levofloxacin doses that could achieve comparable exposures in pregnant participants to those in non-pregnant participants.

## MATERIALS AND METHODS

### Study design

We pooled data from two studies conducted in South Africa. Building Evidence for Advancing new Treatment for Tuberculosis (BEAT Tuberculosis), ClinicalTrials.gov number NCT 04062201, was a phase three randomized controlled trial comparing the efficacy and safety of a novel 6-month treatment regimen for RR-TB with the current 9–12 month South African standard of care regimen conducted in two study sites (19). A subset of adult participants in BEAT Tuberculosis ≥18 years of age underwent pharmacokinetic sampling after approximately 4 weeks of treatment. On the day of the pharmacokinetic visit, a standard breakfast was offered approximately 1 h prior to the observed dose; blood samples were drawn pre-dose and at 2, 4, 6, 8, 10, and 24 h post-dose. A pregnancy pharmacokinetic sub-study within BEAT Tuberculosis was conducted to investigate the effect of pregnancy on drug exposure. Pharmacokinetic sampling (pre-dose, 2, 4, 6, 8, and 24 h post-dose) was performed during the third trimester and repeated 4–8 weeks postpartum.

The second study was a prospective observational cohort study investigating maternal treatment and pregnancy outcomes of women treated for RR-TB at King Dinuzulu Hospital (KDH) in Durban, including an exploration of the effect of pregnancy on plasma concentrations of second-line TB drugs (20). Pregnant women ≥ 18 years of age were recruited for pharmacokinetic analysis, with samples collected during the third trimester and again approximately 6 weeks post-partum. Similarly, participants received a standard breakfast approximately 1 h before an observed dose, and blood samples were drawn pre-dose, 2, 4, and 6 h post-dose.

### Levofloxacin assay

For both studies, EDTA blood samples were centrifuged on site, and plasma samples were stored at −80°C prior to shipping to the University of Cape Town, Division of Clinical Pharmacology Laboratory, where validated total plasma concentration assays were performed using high-performance liquid chromatography with tandem mass spectrometry (HPLC-MS/MS). The lower limit of quantification (LLOQ) for levofloxacin was 0.0781 mg/L (21).

### Population pharmacokinetic modeling

We modeled the levofloxacin concentrations using nonlinear mixed-effects modeling with NONMEM (v7.5.1) (22) and the algorithm first-order conditional estimation with interaction (FOCE-I) to estimate population pharmacokinetic parameters. Perl-speaks-NONMEM (v5.5.6) was employed for the execution of diverse model development and evaluation techniques (23). Pirana (v3.0.0) was utilized to monitor the progress of model development, while Xpose4, in conjunction with RStudio (v1.4.1106), was applied for conducting model diagnostics and post-modeling analysis (23). Several structural models were tested, including one- and two-compartment disposition models with first-order elimination and absorption with and without lag or with transit compartments to model the delay in absorption.

Random effects variability was quantified assuming a log-normal distribution, where between-subject variability (BSV) was tested on disposition parameters and between-occasion variability (BOV) on absorption parameters and bioavailability (24). Due to uncertainty in the timing of the unobserved doses and overall treatment adherence on the days before the PK visits, we investigated the inclusion of additional variability in absorption parameters and bioavailability for unobserved doses. Between-visit variability on clearance was also assessed, as blood samples were collected from most pregnant participants on two separate visits, one antepartum and one postpartum. Further details on the implementation of BSV and BOV, the additional variability for unobserved doses, and the distinction between occasion and visit can be found in the Supplementary material. A combined proportional and additive error was tested and applied to describe the residual unexplained variability (25). Observations below the limit of quantification (BLQ) were imputed to half of the LLOQ in accordance with the M6 method by Beal (26), and their additive error component was inflated by 50% of LLOQ to account for the increased uncertainty in the imputed values, preventing them from disproportionately influencing the model’s fit.

Allometric scaling of clearance (with a fixed exponent of 0.75) and volume of distribution (with a fixed exponent of 1) parameters scaled by either total body weight or fat-free mass (FFM) was investigated to adjust for differences in body size (27). The formula used for calculating FFM (can be found in the Supplementary material) was extended to all participants, including pregnant patients. Although this formula does not fully capture pregnancy-associated changes, it provides a consistent and reproducible approach to adjust for body composition, at least partially. For pregnant participants who were sampled on two separate visits, weight values (and thus FFM) were available at each visit and were handled as time-varying covariates to reflect physiological changes during pregnancy.

After including allometry, other covariate effects were tested, including sex, pregnancy, renal function, HIV status, albumin concentration, co-administration of anti-tuberculosis or antiretroviral drugs (each individually tested as a binary covariate), and study or study site. The effect of pregnancy was tested as categorical: pregnant, postpartum, and never pregnant during the study. Additionally, in pregnant women, we tested the effect of gestational age as a continuous covariate, using linear, exponential, and power functions and centered the effect around the median value in the data set. To investigate the effect of renal function on the pharmacokinetics of levofloxacin, we used serum creatinine concentrations (which were measured close to the pharmacokinetic visit) as a surrogate of glomerular filtration rate in our data. It is reported that creatinine clearance estimation methods have been found to inconsistently underestimate renal function in pregnancy (28) and are therefore not recommended. The effect of serum creatinine on clearance was modeled using a power function with the following formula:


$$CL_{i}=\bar{CL}\cdot {\left(\frac{sCr_{i}}{\bar{sCr\;}}\right)}^{\theta_{sCr}}$$

where ${CL}_{i}$ is the individual clearance in subject *i*, $\bar{CL}$ is the typical value of clearance in the population, *sCr_i_* is the individual serum creatinine in μmol/L, $\bar{sCr\;}$ is the median serum creatinine in the population, and $\theta_{sCr}$ is the exponent of the power function. A linear function and a negative exponential function were also explored and compared based on the drop in objective function value (OFV) and overall fit.

The model building process started by using the structure of a published population PK model of levofloxacin in South African children with multi-drug resistant tuberculosis (MDR-TB) (29), and allometrically scaling disposition parameters to adult body size. The model was first fit on the non-pregnant individuals’ data and, once we obtained a good fit, we added the data with pregnant individuals. Model development was guided by pharmacokinetic modeling principles (30), including statistical significance. The decreases in OFV (ΔOFV) for structural model development and stepwise covariate selection in hierarchical models were presumed to follow a χ2 distribution. This means that a ΔOFV of >3.84 points was deemed significant at *P* < 0.05 for the addition of 1 degree of freedom (df). We also examined diagnostic plots including visual predictive checks, goodness of fit plots, as well as the evaluation of the results for physiological plausibility. The sampling importance resampling procedure (31) was employed to evaluate parameter uncertainty and produce a 95% CI.

### Model-based simulations

Our final model was used to perform Monte Carlo simulations to evaluate exposures achieved using the current WHO dosing guidelines to explore the effects of FFM, serum creatinine, and pregnancy. Due to the small sample size of our study participants (especially pregnant women), we utilized historical data to construct a representative *in silico* population. For non-pregnant TB participants, we sourced information from adult males and females with active TB from five different studies (32–36), in addition to participants in our study. For pregnant women, we used data from the IMPAACT P1078 study (ClinicalTrials.gov number NCT01494038), which included pregnant women with HIV aged ≥18 years in their third trimester receiving isoniazid preventive therapy and exhibited no symptoms of TB (37). We simulated the current WHO guidelines and explored alternative dosing scenarios for pregnant women. We calculated the AUC_0-24_ that would achieve the proposed *f*AUC_0-24_/MIC efficacy target of 146 from the hollow-fiber system model of tuberculosis (38) using the reported levofloxacin wild-type MIC value of 0.5 mg/L (39). We then compared our observed and simulated AUC_0-24_ and C_max_ values to those reported in other levofloxacin studies in adults with drug-resistant TB (14, 40–43) with AUC_0-24_ and C_max_ range of medians of 98.8–145 mg·h/L and 7.40–14.86 mg/L respectively, detailed in Table S2.

In addition to exploring the exposures in the *in silico* population described above, we repeated the simulations in some chosen representative typical patients in each weight band (based on the values and correlations between weight, height, and serum creatinine in our study data shown in Fig. S3). Serum creatinine values were categorized into “low,” “medium,” and “high” levels using tertiles within each group of participants, based on the weight, sex, and pregnancy status. This tertile-based approach divides values into three equal parts, allowing for a data-driven classification that reflects the distribution of serum creatinine within each subgroup.

## RESULTS

### Study population and data

Table 1 shows the participants’ characteristics stratified by study and subpopulations in our data set. We included a total of 47 participants from the two studies, 33 (70%) females and 38 (81%) black, with median (range) age of 32 (19–51) years, weight 58.0 (37.0–98.0) kg, serum creatinine 56.2 (25.3–110) µmol/L, and serum albumin 30.5 (17.0–40.0) g/L. As expected, serum creatinine was found to be lower in pregnant participants during the third trimester of pregnancy compared with postpartum concentrations and also lower compared to men and non-pregnant women. Of 47 participants, 31 (66%) were living with HIV and on antiretroviral therapy (ART), commonly with dolutegravir-based ART (*n* = 15, 48%). Of the 33 female participants included, 21 (64%) were pregnant, with 12 (57%) of these contributing matched antepartum and postpartum profiles. We analyzed a total of 19 antepartum, 14 postpartum, 12 non-pregnant female, and 14 male pharmacokinetic profiles. A total of 320 levofloxacin concentration samples were available for analysis, including 6 (1.88%), which were BLQ (all at pre-dose).

**TABLE 1:** Characteristics of study data participants treated for rifampicin-resistant tuberculosis^*a*^

	BEAT tuberculosis study				KDH study		Overall participants
	Antepartum (*n* = 6)	Postpartum (*n* = 6)	Non-pregnant females (*n* = 12)	Males (*n* = 14)	Antepartum (*n* = 13)	Postpartum (*n* = 8)	(*n* = 47)
Age (years)	31 (19–42)	30 (19–38)	34 (20–45)	38 (27–51)	30 (23–48)	31 (23–48)	32 (19–51)
Weight (kg)	62.9 (58.2–77.5)	59.1 (49.8–67.2)	52.9 (37.0–67.9)	54.4 (41.9–64.0)	60.0 (43.0–98.0)	56.0 (45.0–83.5)	58.0 (37.0–98.0)
Height (m)	1.56 (1.50–1.60)	1.58 (1.50–1.60)	1.57 (1.46–1.80)	1.73 (1.58–1.88)	1.63 (1.51–1.77)	1.56 (1.52–1.65)	1.60 (1.46–1.88)
Fat-free mass (kg)	38.3 (37.6–43.4)	37.8 (33.5–40.3)	35.7 (27.3–45.3)	46.4 (37.9–51.2)	39.0 (31.7–55.2)	36.1 (30.8–46.2)	39.4 (27.3–51.2)
Serum creatinine (µmol/L)	43.1 (37.2–54.0)	57.3 (52.2–60.5)	58.5 (37.5–110)	64.1 (52.3–102)	47.5 (37.0–66.4)	53.5 (25.3–63.0)	56.2 (25.3–110)
Serum albumin (g/L)	26.0 (25.0–30.0)	32.0 (28.0–35.0)	29.0 (21.0–40.0)	29.0 (17.0–38.0)	29.0 (23.0–31.0)	33.5 (14.0–37.0)	30.5 (17.0–40.0)
Race, n (%)							
Black	4 (67%)	4 (67%)	7 (58%)	12 (86%)	13 (100%)	8 (100%)	38 (81%)
White	2 (33%)	2 (33%)	5 (42%)	2 (14%)	0 (0%)	0 (0%)	9 (19%)
HIV positive, n (%)	5 (83%)	5 (83%)	7 (58%)	6 (43%)	11 (85%)	7 (88%)	31 (66%)
ART, n (%)							
TDF–FTC–EFV	1 (20%)	2 (40%)	1 (14%)	3 (50%)	0 (0%)	0 (0%)	6 (19%)
TDF–3TC–DTG	4 (80%)	3 (60%)	5 (71%)	2 (33%)	4 (36%)	3 (43%)	15 (48%)
TDF–3TC–NVP	0 (0%)	0 (0%)	0 (0%)	1 (17%)	0 (0%)	0 (0%)	1 (3%)
TDF–FTC–LPV/r	0 (0%)	0 (0%)	1 (14%)	0 (0%)	0 (0%)	0 (0%)	1 (3%)
TDF–FTC–NVP	0 (0%)	0 (0%)	0 (0%)	0 (0%)	7 (64%)	4 (57%)	7 (23%)
Most common TB co-treatment, n (%)							
LNZ	4 (67%)	4 (67%)	12 (100%)	14 (100%)	10 (77%)	2 (25%)	40 (85%)
DLM	2 (33%)	2 (33%)	5 (42%)	5 (36%)	1 (8%)	0 (0%)	13 (28%)
CLF	6 (100%)	6 (100%)	12 (100%)	14 (100%)	13 (100%)	8 (100%)	47 (100%)
BDQ	5 (83%)	5 (83%)	12 (100%)	14 (100%)	13 (100%)	7 (88%)	44 (94%)

^*a*^ Data are expressed as median (range) or number (percent). ART, antiretroviral therapy; DTG, dolutegravir; TDF, tenofovir disoproxil fumarate; FTC, emtricitabine; EFV, efavirenz; 3TC, lamivudine; NVP, nevirapine; LPV/r, lopinavir boosted with ritonavir; LZD, linezolid; DLM, delamanid, CLF, clofazimine; BDQ, bedaquiline. Note: BEAT Tuberculosis study and KDH study contributed five and seven matched pre- and postpartum profiles, respectively. Non-pregnant females refer to women who were not pregnant during the study.

### Population pharmacokinetic modeling

Levofloxacin pharmacokinetics was well described by a one-compartment model with first-order elimination and first-order absorption with transit compartments, as illustrated in Fig. S1. Addition of a second compartment did not improve the fit (∆OFV, 0.674; 2 df; *P* > 0.05). Absorption transit compartments, whose number was fixed to 20 to improve model stability after a sensitivity analysis, significantly improved the fit compared with using a lag time (∆OFV, 5.87; 1 df; *P* < 0.05). Inclusion of allometric scaling with FFM mass also significantly improved the model fit (∆OFV, 12.9) and performed better than using total body weight (∆OFV, 3.46). Table 2 shows the final parameter estimates including precision. The model estimated that, for a non-pregnant participant with a FFM of 39.4 kg and serum creatinine of 56.2 µmol/L, the typical value of clearance was 6.06 L/h, and the volume of distribution was 85.9 L. Higher serum creatinine levels were associated with lower levofloxacin clearance using a power function with an exponent of −0.367 (∆OFV, 6.07; 1 df; *P* < 0.05), as illustrated in Fig. S2. After accounting for the effect of body size and serum creatinine, pregnancy was found to further increase levofloxacin clearance by 38.1% (∆OFV, 58.4; 1 df; *P* < 0.001). In a model without accounting for serum creatinine effect, shown in Table S3, the effect of pregnancy on levofloxacin clearance was larger (53%).

**TABLE 2:** Pharmacokinetic parameter values of levofloxacin in adults with rifampicin-resistant tuberculosis

PK parameter	Typical value (95% CI)^*a*^
Clearance, CL (L/h)^*b*^	6.06 (5.47–6.53)
Volume of distribution, V (L)^*b*^	85.9 (80.6–91.7)
Mean transit time, MTT (h)	1.07 (0.771–1.32)
Number of transit compartments, NN (.)	20 (fixed)
Absorption rate constant, Ka (1 /h)	1.59 (1.11–2.40)
Bioavailability, *F* (.)	1 (fixed)
Effect of pregnancy on CL (%)	+38.1 (+23.4 – +57.1)
Effect of serum creatinine on CL (power exponent)	−0.367 (-0.493–-0.104)
Scaling factor for BOV on *F* for unobserved doses (-fold)^*c*^	2.35 (1.68–3.28)
Additive error (mg/L)	0.244 (0.130–0.394)
Proportional error (%)	7.33 (6.49–8.10)
	
Between-subject variability (%)^*d*^	
CL	22.1 (17.1–28.3)
	
Between-occasion variability (%)^*d*^	
MTT	45.9 (30.5–70.3)
Ka	85.6 (61.7–119)
*F*	23.7 (19.3–28.1)

^*a*^ 95% confidence intervals obtained with sampling importance resampling technique.

^*b*^ This parameter has been adjusted by allometric scaling, and the values reported here refer to a subject with a fat-free mass of 39.4 kg.

^*c*^ This is a multiplicative factor increasing the between-occasion variability in bioavailability for all predose concentrations, which follow an unobserved dose.

^*d*^ Between-subject variability and between-occasion variability were assumed to be log-normally distributed and reported as the percent coefficient of variation (CV) calculated as:$\%CV=\sqrt{\omega^{2}}\cdot 100$

Finally, pre-dose concentrations exhibited greater variability than expected compared with post-dose concentrations, likely due to inaccurate information regarding unobserved doses prior to the pharmacokinetic visit. The model accounted for this by estimating ~2.35-fold larger between-occasion variability in bioavailability (was more significant than in the absorption parameters) for unobserved doses attached to pre-dose concentrations (∆OFV, 16.4; 1 df, *P* < 0.001). Addition of between-visit variability on clearance was not significant and therefore not retained in the model. Other covariates examined, including sex, HIV status, gestational age, concomitant medication, albumin concentrations, study (BEAT Tuberculosis vs KDH study), and study sites had no significant effect on the pharmacokinetics of levofloxacin.

**Fig 1:**
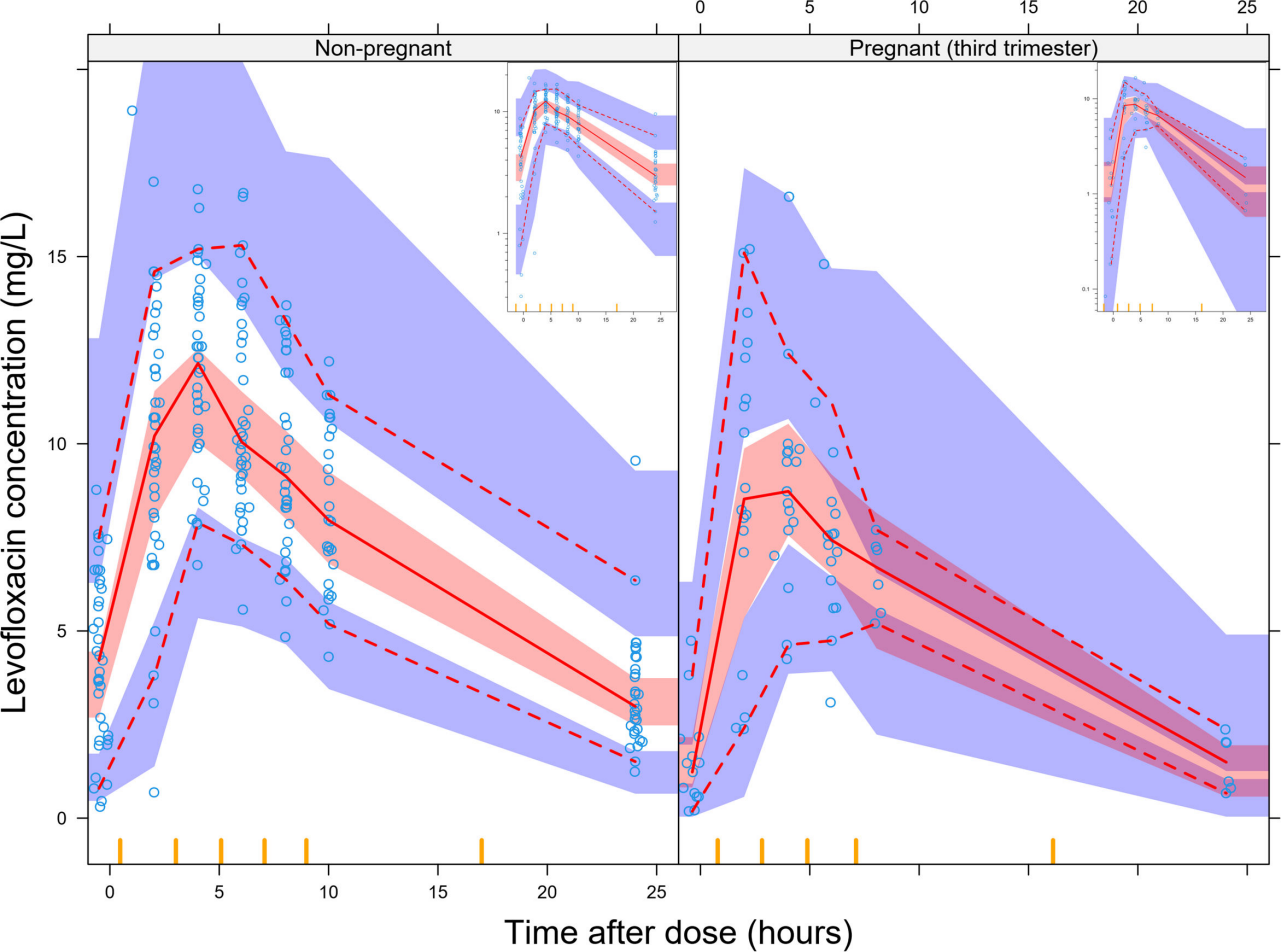
Visual predictive check (VPC) of levofloxacin concentration versus time after dose stratified by pregnancy status (the non-pregnant stratum comprises the postpartum, females not pregnant during the study, and male concentrations). The solid and dashed lines represent the 50th, 5th, and 95th percentiles of the observed data, while the shaded areas represent the model-predicted 95% confidence intervals for the same percentiles. The blue circles are the observed concentrations. The inset figures are the same plots plotted in the log scale.

Our model adequately fit the data as depicted by the visual predictive check (VPC) shown in Fig. 1. The stratification of the VPC by pregnancy status confirms lower median exposures in the pregnant stratum compared with the non-pregnant stratum.

Fig. S4 shows the observed AUC_0-24_ and C_max_ in the different participant groups in our data. Overall, the median (interquartile range [IQR]) AUC_0-24_ and C_max_ observed were 131 (108–170) mg⋅h/L and 11.3 (9.68–13.7) mg/L respectively. Levofloxacin exposures in pregnant women during the antepartum period were generally lower than those observed in the same women postpartum, as well as in males and non-pregnant females.

### Model-based simulation results

**Fig 2:**
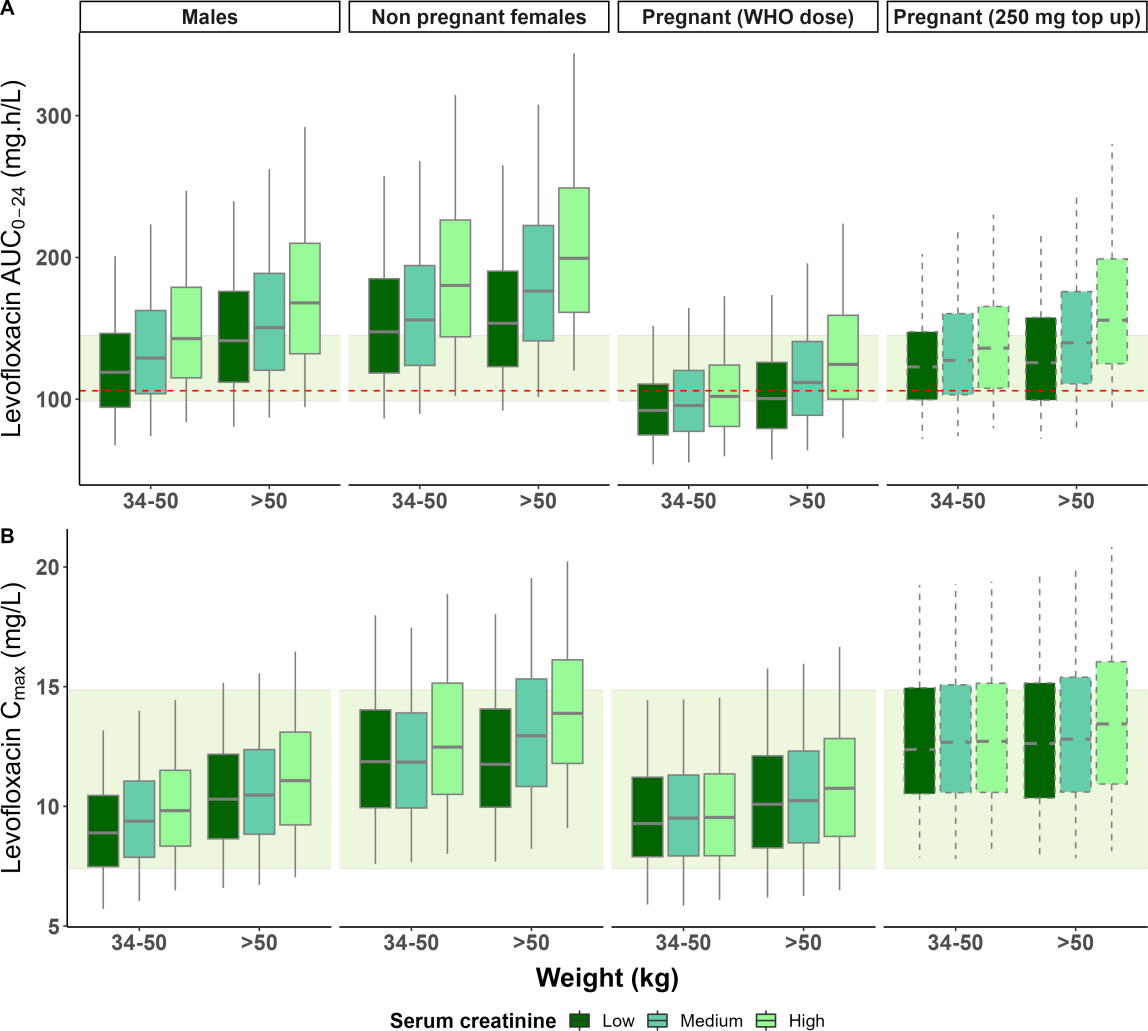
Simulated levofloxacin steady-state AUC_0-24_ (**A**) and C_max_ (**B**) versus weight with different levels of serum creatinine (categorized into “low,” “medium,” and “high” using tertiles within each group of participants, based on the weight, sex, and pregnancy status) in the historical data. The shaded ribbon represents the range of the reported adult median AUC_0-24_ (98.8–145 mg.h/L) and C_max_ (7.40–14.86 mg/L) from literature with similar dosing strategy for adults (750–1,000 mg based on body weight) with drug-resistant TB (14, 40–43). The boxes indicate the interquartile range, while the whiskers denote the 2.5th and 97.5th percentiles. The dashed boxplots represent the proposed additional 250 mg once-daily dose exposure for the pregnant women in the third trimester. The horizontal red dashed line represents the AUC_0-24_ of 106 mg·h/L calculated based on the proposed *f*AUC_0-24_/MIC efficacy target of 146 from the hollow-fiber system model of tuberculosis (38) using reported levofloxacin wild-type MIC of 0.5 mg/L (39) and assuming an unbound fraction (*fu)* of 69% (12, 44) using the following equation: ${AUC}_{0-24}\geq \frac{146∙MIC}{fu}.$ Non pregnant females refer to women who were not pregnant during the study.

The results of the Monte Carlo simulations using the historical *in silico* population of pregnant and non-pregnant adults are shown in Fig. 2, while those using representative typical patients from our study data are shown in Fig. S5 (a summary of the historical data compared to the study data is shown in Table S1; Fig. S6). The model predicts that the AUC_0-24_ and C_max_ achieved in pregnant women on the current WHO recommended doses are consistently lower than their non-pregnant female counterparts and males. Non-pregnant females’ exposures are predicted to be higher than the males, which was also seen in the individual values from our final model shown in Fig. S4. Participants in the higher weight band are predicted to have higher levofloxacin exposures, as are individuals with higher serum creatinine levels. Overall, the median simulated exposures in non-pregnant individuals are in line with the reported range of median AUC_0-24_ and C_max_ in adults with drug-resistant TB. The values of AUC in most of these subjects are also predicted to be larger than 106 mg⋅h/L, the value corresponding to the efficacy target *f*AUC_0-24_/MIC = 146 (38) when using the levofloxacin wild-type MIC of 0.5 mg/L (39). Our model predicts that for pregnant women to achieve exposures comparable to their non-pregnant counterparts, a dose increase of 250 mg daily is required across all the weight bands under study (34–50 kg and above 50 kg).

## DISCUSSION

We developed a population pharmacokinetic model describing levofloxacin in adults with RR-TB and characterized the effect of pregnancy. We found that levofloxacin clearance increases with body size and renal function, both of which are larger in pregnancy. After adjusting for these factors, we found a further 38% increase in levofloxacin clearance during the third trimester. This leads to significantly lower levofloxacin exposures in pregnancy, which is a concern, since levofloxacin is a group A drug recommended for inclusion in all RR-TB treatment regimens (3), and it is being increasingly explored as a drug for TB prophylaxis (9–11). Our Monte Carlo simulations suggest that increasing the daily dose of levofloxacin by 250 mg in pregnant women would compensate for their increased clearance.

Our findings are in line with our knowledge of the pharmacokinetics of levofloxacin and the pregnancy-related physiological changes that could affect its concentrations (18, 45). Since levofloxacin is mainly excreted unchanged in urine (12), elimination is closely tied to the increased renal function in pregnancy. The effect of serum creatinine was significant in the model even when we only included non-pregnant participants, and it explained part of the variability between individuals. Pregnant women typically exhibit lower serum creatinine concentrations (18, 46–48), and this was the case in our cohort, with median serum creatinine 45.3 µmol/L during the third trimester and 55.4 µmol/L during the postpartum visit, compared with 58.5 µmol/L in non-pregnant women.

Besides renal function, we identified body size as significantly affecting the disposition of levofloxacin, i.e., larger individuals had larger clearance and volume of distribution, in line with the well-established principle of allometry (27). More specifically, we observed that exposures in non-pregnant females (women who were not pregnant during the study) were higher than in males, even though they had similar total body weight. This effect was explained in the model by using FFM as the best size descriptor for the disposition parameters. Women’s bodies are known to have a larger proportion of essential body fat, and hence a lower proportion of metabolically active body mass (49), thus resulting in lower clearance for many drugs, including levofloxacin. This means that, while the effect of sex *per se* was not explicitly added in the model (as it was not found to be statistically significant), the influence of sex was indirectly mediated through other covariates such as FFM (and serum creatinine), which differ between males and females. This finding is in line with a recent review paper on inclusion of covariate effects in pharmacometrics modeling, highlighting that, after accounting for the influence of primary covariates like body size and composition, renal function, and genetics, the impact of covariates such as race and sex is typically negligible or non-existent (50).

Pregnant women experience increased total body weight, total fat mass, and total body water (16, 45), and our cohort was no exception: median total body weight and FFM of 61.5 and 38.7 kg during the third trimester, 57.6 and 37 kg during the postpartum visit, compared with 52.9 and 35.7 kg in non-pregnant women. In our model, allometric scaling was based on FFM and not total body weight, so the pregnancy-related increase in weight was related only partly to an increase in FFM, and the resulting increase in clearance is less than the raw change in total body weight would suggest. After including allometry, we tested for an effect of pregnancy on the volume of distribution but did not find a statistically significant effect.

As discussed above, pregnant women are characterized by both lower serum creatinine and larger body size, so both factors contributed to an increase in levofloxacin clearance. In our analysis, after accounting for the FFM- and serum creatinine-mediated changes, we identified a further increase in clearance of 38.1%. It is unclear whether this additional increase is due to other separate pregnancy-related physiological changes, or rather to the fact that our model is unable to adequately account for changes in FFM and renal function during pregnancy (as discussed in the limitation section below). Pregnancy is known to also affect the protein concentrations in plasma proteins, in particular decreasing albumin (51). While such a change may influence the pharmacokinetics of highly protein-bound drugs, levofloxacin is only moderately bound (24%–38%) to plasma proteins (12). In our study, albumin concentrations were similar between pregnant and non-pregnant participants, and albumin concentration was not found to significantly affect levofloxacin clearance. Finally, pregnancy is known to cause changes in the expression and/or activity of drug-metabolizing enzymes (52), but this is unlikely to be a major contributing factor, since levofloxacin undergoes minimal metabolism (<5%) (12).

In summary, it is important to note that, in our analysis, the overall effect of pregnancy is “divided” between the increase caused by renal function, the increase caused by body size, and finally the additional “pregnancy” effect (+38% in clearance). Numerically, the overall clearance increase due to pregnancy was observed to be around 53%, which is the value we obtained when we used an alternative model (in Supplementary material) that did not adjust for serum creatinine. Despite arguably being more complex and difficult to understand, we selected as our final model the version including the effect of serum creatinine. This was not only because it provided the best fit to the data but also because it offered a more physiologically plausible explanation of the observed differences not only between pregnant and non-pregnant women, but also within non-pregnant participants. A further advantage of this more complex model was the fact that, without including any specific effect for the postpartum cohort, it was able to predict that the levofloxacin exposures in the postpartum visit were larger than during pregnancy and similar to those in men but still lower than those observed in non-pregnant women. This is because the pregnancy-related physiological changes in serum creatinine levels and body weight might not have fully returned to the pre-pregnancy state.

Our findings are consistent with previous studies on levofloxacin pharmacokinetics in non-pregnant adults. Our model structure aligns with those by Sidamo et al. (42) and Elsen et al. (43) who found that a one-compartment disposition model best described levofloxacin pharmacokinetics in MDR-TB patients. Kervezee et al. reported similar results in healthy subjects (53). In contrast, Boonpeng et al. (54) identified a two-compartment model in healthy volunteers, but the sum of their central and peripheral volumes of distribution is comparable to our volume of distribution, suggesting a similar overall distribution. Differences in sampling schedules may explain the identification of a second compartment in their study. Our estimates of clearance and volume of distribution are also in line with other adult studies in TB patients (43) and patients with lower respiratory tract infections (55).

The observed levofloxacin exposures in our study, with median (IQR) AUC_0-24_ 131 (108–170) mg⋅h/L and C_max_ 11.3 (9.68–13.7) mg/L, are comparable to exposures reported in similar MDR-TB studies. Mohammed et al. (41) reported median (IQR) AUC_0-24_ 140 (102–179) mg⋅h/L and C_max_ 14.4 (9.8–16.4) mg/L in Tanzanian adults with RR-TB, while Peloquin et al. (40) observed median (IQR) AUC_0-24_ 145 (103–457) mg⋅h/L and C_max_ 14.9 (9.89–29.2) mg/L in South African and Peruvian adults. The calculated AUC_0-24_ of 106 mg⋅h/L, targeting an *f*AUC_0-24_/MIC efficacy threshold of 146 (38), falls within the observed and simulated AUC_0-24_ range in our population, aligning with the reported *f*AUC/MIC efficacy target of ≥100 (56).

We also found that, with the current dosing recommendations, participants in higher weight bands achieve higher levofloxacin exposures. This finding is not surprising, since levofloxacin is dosed using weight bands targeting a constant mg/kg dose across the weight spectrum. This does not suitably account for the nonlinear nature of allometry and is known to cause lower exposure in smaller individuals, as explained in Denti et al. (57).

Our study has some limitations. First, our sample size of pregnant participants was small, and we only had data in the third trimester. We were therefore unable to determine whether earlier stages of pregnancy also affect levofloxacin pharmacokinetics. Second, serum creatinine measurements were not always obtained on the exact pharmacokinetic visit (±2 weeks), potentially introducing variability in renal function estimates. Additionally, since no validated renal function estimation formula currently exists for pregnancy, our model should be used with caution when extrapolating to serum creatinine levels outside of those observed in our study. On the other hand, our model was able to predict levofloxacin clearance in patients with severely impaired kidney function (58) as shown in the Supplementary material. Similarly, while we used an FFM formula to estimate body composition in all participants, including pregnant women, a more pregnancy-specific approach requiring total body water measurements (59) was not available in our study. While the formula may not be highly accurate during pregnancy, it is a reproducible method. Finally, due to limited data on pregnant women with active TB, our simulations included historical data from patients receiving TB preventive therapy—this was supported by the fact that levofloxacin is increasingly used in TB prophylaxis contexts (9–11).

To achieve levofloxacin exposures comparable to non-pregnant participants receiving the standard 750–1,000 mg once daily dose, our simulations suggest that pregnant women would require a 250 mg dose increase. We recommend that this dose adjustment be considered during the third trimester. At the current dose levels, RR-TB treatment in pregnant women is safe for both the mother and her infant (20). However, the safety of higher levofloxacin doses in pregnancy, particularly concerning fluoroquinolone-associated toxicities, such as tendinitis, tendon rupture (60), peripheral neuropathy (61), and potential to cause QTc prolongation (62) require further investigation.

In conclusion, pregnant women achieve lower exposures of levofloxacin at the currently recommended doses of 750–1,000 mg once daily compared with non-pregnant individuals. Increasing the dose by an additional 250 mg once daily restores exposure to levels observed in non-pregnant participants and should be considered as a dosing strategy. Further studies are required to assess the safety implications of our proposed adjustment.

## Data Availability

The data that support the findings of this study are available from the corresponding author upon reasonable request.

## AFTER EPUB

*[This article was published on 1 April 2025 with an error in a Table 2 footnote. The footnote was corrected in the current version, posted on 17 April 2025.]*

## SUPPLEMENTAL MATERIAL

Supplemental material aac.01626-24-s0001.docx Tables S1 to S3, Fig. S1 to S7, and further description of some methods, model code, and snapshot of dataset.

ASM does not own the copyrights to Supplemental Material that may be linked to, or accessed through, an article. The authors have granted ASM a non-exclusive, world-wide license to publish the Supplemental Material files. Please contact the corresponding author directly for reuse.
