# Supplementary material for: The effect of pregnancy on the population pharmacokinetics of levofloxacin in South Africans with rifampicin-resistant tuberculosis
Source: Antimicrob Agents Chemother. 2025 Apr 1;69(5):e01626-24. doi: 10.1128/aac.01626-24 (PMC12057369; doi:10.1128/aac.01626-24)
Supplement: Supplemental material — Tables S1 to S3, Fig. S1 to S7, and further description of some methods, model code, and snapshot of dataset. [file aac.01626-24-s0001.docx]

**Supplementary material**

**Table S1:** Characteristics of study data in comparison to historical data

|  | **Historical data** | | | **Study data** | | | **Overall participants** |
| --- | --- | --- | --- | --- | --- | --- | --- |
|  | **Pregnant**  **(3rd trimester)**  **(n = 307)** | **Non-pregnant females**  **(n = 232)** | **Males**  **(n = 274)** | **Pregnant**  **(3rd trimester)**  **(n = 19)** | **Non-pregnant females**  **(n = 12)** | **Males**  **(n = 14)** | **(n = 858)** |
| **Age (years)** | 29 (18-44) | 31 (18-69) | 36 (18-77) | 30 (19-48) | 34 (20-45) | 38 (27-51) | 32.5 (18-77) |
| **Weight (kg)** | 65.6 (45.1-109) | 54.0 (28.0-111) | 54.0 (34.4-105) | 61.5 (43.0-98.0) | 52.9 (37.0-67.9) | 54.4 (41.9-64.0) | 54.2 (28.0-111) |
| **Height (m)** | 1.57 (1.13-1.76) | 1.57 (1.15-1.77) | 1.70 (1.46-1.88) | 1.60 (1.50-1.77) | 1.57 (1.46-1.80) | 1.73 (1.58-1.88) | 1.59 (1.13-1.88) |
| **Fat-free mass (kg)** | 39.7 (28.0-55.6) | 34.9 (21.7-55.7) | 46.4 (34.7-73.2) | 38.7 (31.7-55.2) | 35.7(27.3-45.3) | 46.4 (37.9-51.2) | 39.2 (21.7-73.2) |
| **Serum creatinine (µmol/L)** | 42.4 (17.7-79.6) | 59.0 (40.0-114) | 67.3 (40.0-119) | 45.3 (37.0-66.4) | 58.5 (37.0-110) | 64.1 (52.3-102) | 58.8 (17.7-119) |
| **Race n (%)** |  |  |  |  |  |  |  |
| **Black** | 282 (92%) | 85 (37%) | 75 (27%) | 17 (89%) | 7 (58%) | 12 (86%) | 478 (56%) |
| **White** | 0 (0%) | 34 (15%) | 38 (14%) | 2 (11%) | 5 (42%) | 2 (14%) | 81 (9%) |
| **Asian** | 25 (8%) | 65 (28%) | 108 (39%) | 0 (0%) | 0 (0%) | 0 (0%) | 198 (23%) |
| **Mixed race** | 0 (0%) | 48 (21%) | 53 (19%) | 0 (0%) | 0 (0%) | 0 (0%) | 101 (12%) |
| **HIV positive n (%)** | 307 (100%) | 98 (42%) | 117 (43%) | 16 (84%) | 7 (58%) | 6 (43%) | 551 (64%) |

Data are expressed as median (range) or number (percent). Non-pregnant females in both cohorts refer to the women who were not pregnant during the study excluding postpartum data)

**Table S2:** Summary of reported levofloxacin AUC_0-24_ and C_max_ in adults with tuberculosis (TB)

|  |  |  |  |  |  |  |  |
| --- | --- | --- | --- | --- | --- | --- | --- |
| **Study** | **Sample size (n)** | **TB type** | **Age (yrs.)** | **Weight (kg)** | **LVF dose** | **AUC_0-24_ (mg⋅h/L)** | **C_max_ (mg/L)** |
| ^1^ | 20 | MDR/XDR | 31 (27-35) ** | 63.35 (55.25-77) ** | 15mg/kg (750-1000 mg) | 98.8 (84.4-159.6) ** | 10.05 (8.4-16.2) ** |
| ^2^ | 26 | MDR-TB | 28 (18-60) * | 52 (40-75) * | 17 mg/kg (750-1000 mg) | 145 (103-457) * | 14.86 (9.89-29.17) * |
| ^3^ | 45 | MDR-TB | 39 (32-45.5) ** | 51 (46.5-61) ** | 750-1000 mg | 140 (102.4-179.1) ** | 14.4 (9.8-16.4) ** |
| ^4^ | 21 | MDR-TB | 26 (20-30) ** | 45 (42.5-52.3) ** | 1000 mg | 103.4 (71.5-108.6) ** | 7.4 (6.1-9.4) ** |
| ^5^ | 30 | MDR-TB | 41.5 (33.5-48.0) ** | 54.6 (47.9-59.9) ** | 750-1000 mg | 129 (118-191) ** | 10.3 (7.9-15.4) ** |

*Data are expressed as median (range), **Data are expressed as median (interquartile range; (IQR)); MDR, Multidrug-resistant; XDR, extensively drug-resistant; LVF, levofloxacin; AUC_0-24_, Area Under the Concentration time curve observed over 24 hours; C_max_, maximum concentration.

**Table S3:** Pharmacokinetic (PK) parameter values of levofloxacin without the effect of serum creatinine on clearance

|  |  |
| --- | --- |
| **PK parameter** | **Typical value (95% CI)^a^** |
| Clearance, CL (L/h)^b^ | 5.95 (5.46 – 6.52) |
| Volume of distribution, V (L)^b^ | 86.1 (80.0 – 92.1) |
| Mean transit time, MTT (h) | 1.08 (0.802 – 1.34) |
| Number of transit compartments, NN (.) | 20 (fixed) |
| Absorption rate constant, Ka (1/h) | 1.61 (1.11 – 2.31) |
| Bioavailability, *F* (.) | 1 (fixed) |
| Effect of pregnancy on CL (%) | +53.0 (+39.0 – +69.4) |
| Scaling factor for BOV on *F* for unobserved doses (-fold)^c^ | 2.41 (1.64 – 3.44) |
| Additive error (mg/L) | 0.234 (0.125 – 0.374) |
| Proportional error (%) | 7.38 (6.16 – 8.57) |
|  |  |
| Between-subject variability (%)^d^ |  |
| CL | 24.7 (19.3 – 30.9) |
|  |  |
| Between-occasion variability (%)^d^ |  |
| MTT | 45.5 (31.9 – 65.8) |
| Ka | 85.7 (63.9 – 116) |
| *F* | 23.4 (19.2 – 29.5) |
|  |  |

^a^95% confidence intervals obtained with sampling importance resampling technique.

^b^This parameter has been adjusted by allometric scaling, and the values reported here refer to a subject with a fat-free mass of 39.4 kg.

^c^This is a multiplicative factor increasing the between-occasion variability in bioavailability for all predose concentrations, which follow an unobserved dose.

^d^Between-subject variability and between-occasion variability were assumed to be log-normally distributed and reported as the percent coefficient of variation (CV) calculated as $\%CV= \sqrt{\omega^{2}}\cdot100$.


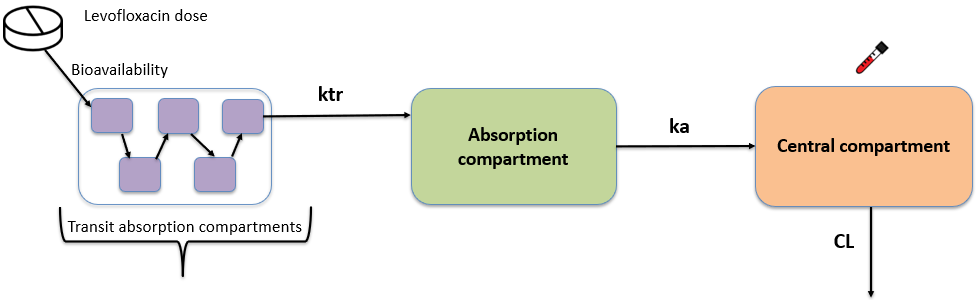


**Figure S1:** Levofloxacin pharmacokinetic model structure. Ktr: transit rate constant; ka: absorption rate constant; CL: levofloxacin clearance.


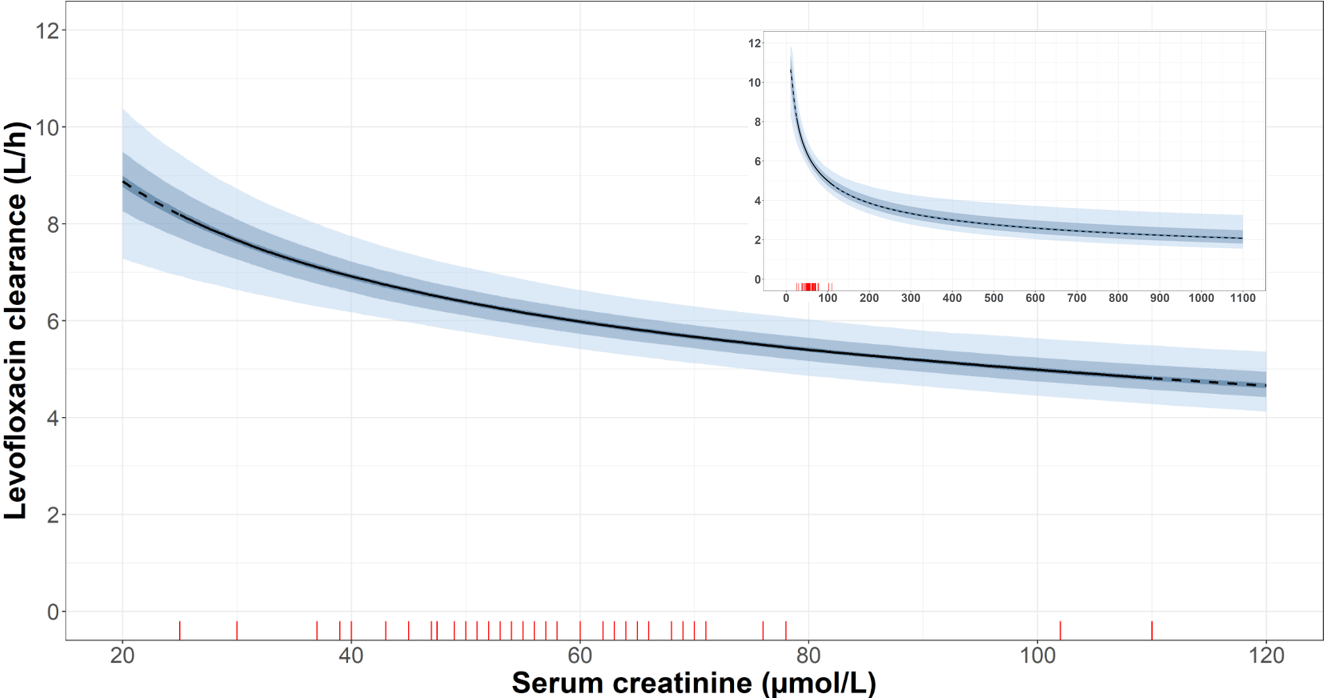


**Figure S2:** Relationship between levofloxacin clearance and serum creatinine levels using a power function:

${CL}_{i}=\bar{CL}\cdot\left( \frac{{sCr}_{i}}{\bar{sCr}} \right)^{\theta_{sCr}}$where ${CL}_{i}$ is the individual clearance in subject *i*, $\bar{CL}$ is the typical value of clearance in the population, *sCr_i_* is the individual serum creatinine in $\mu mol/L$, $\overline{sCr}$ is the median serum creatinine in the population, and $\theta_{sCr}$ is the exponent of the power function. The solid black lines and the red tick marks on the x axis show the observed serum creatinine values in our cohort while the dashed lines show extrapolation to extreme values (zoomed in the inset figure), based on observed serum creatinine in hemodialysis patients reported by Tsuruoka et al^6^. The shaded areas represent the 10^th^ ,50^th^ and 90^th^ prediction intervals of the predicted levofloxacin clearance.


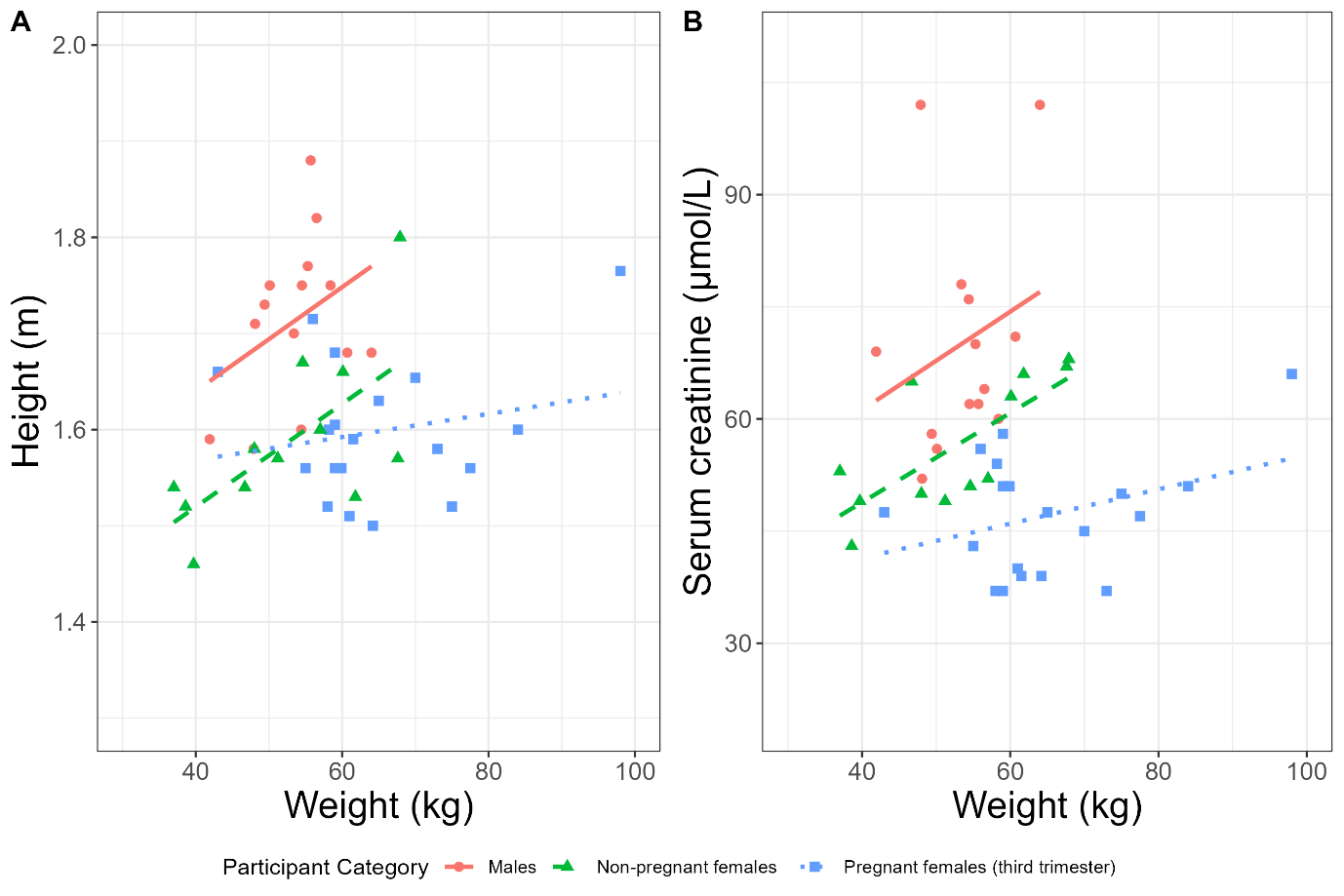


**Figure S3:** Scatter plot of height versus weight (A) and serum creatinine versus weight (B) based on values in our study data. It shows a positive correlation between height, weight and serum creatinine which was considered when choosing the representative typical patients from the study data for additional simulations. Non-pregnant females refer to women who were not pregnant during the study.


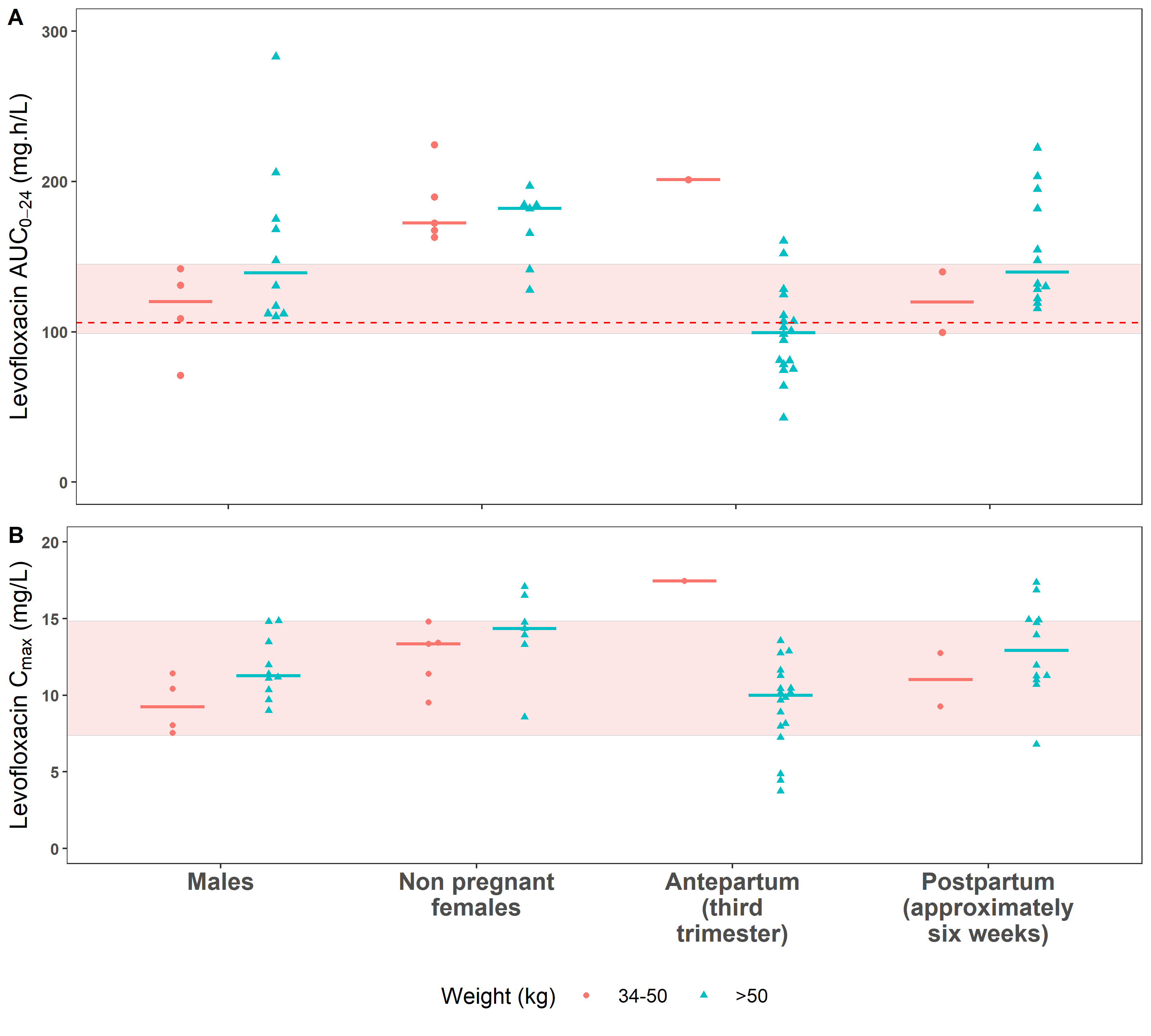


**Figure S4:** Levofloxacin steady-state AUC_0-24_ (panel A) and C_max_ (Panel B) versus weight based on individual values from our final model for all the participant groups in our study data. The shaded ribbon represents the range of the reported adult median AUC_0-24_ (98.8-145 mg⋅h/L) and C_max_ (7.40-14.86 mg/L) from literature with similar dosing strategy for adults (750-1000 mg based on body weight) with drug-resistant TB^1–5^. The horizontal red dashed line represents the AUC_0-24_ of 106 mg⋅h/L calculated based on the proposed *f*AUC_0-24_/MIC efficacy target of 146 from the hollow fibre system model of tuberculosis^7^ using reported levofloxacin wildtype MIC of 0.5 mg/L^8^ and assuming an unbound fraction (*fu)* of 69%^9^ using the following equation: ${AUC}_{0-24} \geq\frac{146\cdot MIC}{fu}.$ Non pregnant females refer to women who were not pregnant during the study.


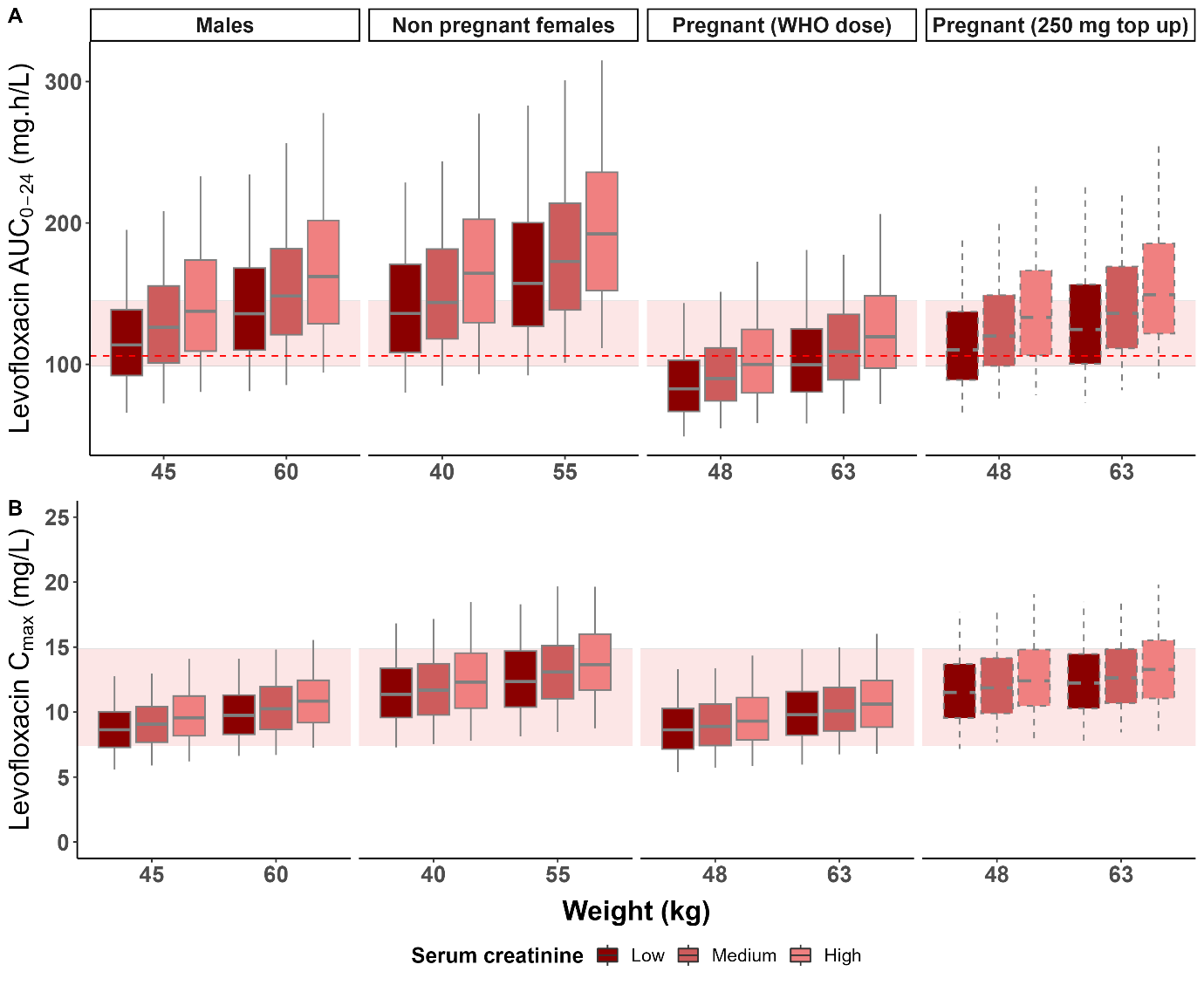


**Figure S5:** Simulated levofloxacin steady-state AUC_0-24_ (panel A) and C_max_ (Panel B) versus weight with three levels of serum creatinine (categorized into “low”, “medium” and “high” using tertiles within each group of participants, based on the weight, sex and pregnancy status) for the chosen representative typical patients in our study data. The shaded ribbon represents the range of the reported adult range of median AUC_0-24_ (98.8-145 mg.h/L) and C_max_ (7.40-14.86 mg/L) from literature with similar dosing strategy for adults (750-1000mg based on body weight) with drug-resistant TB^1–5^. The boxes indicate the interquartile range, while the whiskers denote the 2.5^th^ and the 97.5^th^ percentiles. The dashed boxplots represent the proposed additional 250mg once daily dose exposure for the pregnant women in the third trimester. The horizontal red dashed line represents the AUC_0-24_ of 106 mg⋅h/L calculated based on the proposed *f*AUC_0-24_/MIC efficacy target of 146 from the hollow fibre system model of tuberculosis^7^ using reported levofloxacin wildtype MIC of 0.5 mg/L^8^ and assuming an unbound fraction (*fu)* of 69%^9^ using the following equation: ${AUC}_{0-24} \geq\frac{146\cdot MIC}{fu}.$ Non pregnant females refer to women who were not pregnant during the study.


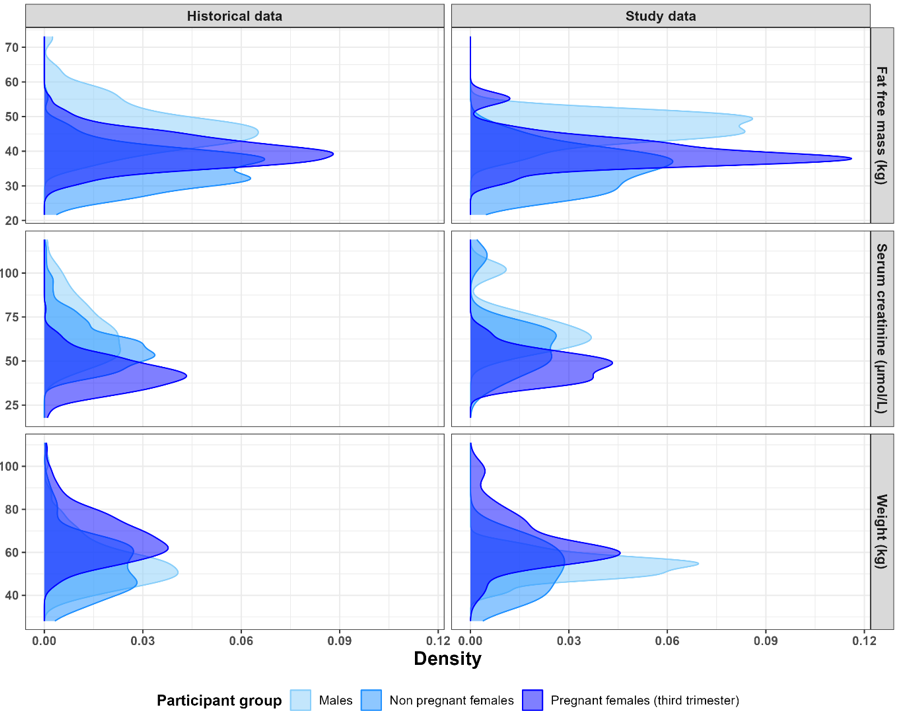


**Figure S6:** Density plots for the historical data versus our study data showing the distribution of variables used in our levofloxacin pharmacokinetic model. Non pregnant females refer to women who were not pregnant during the study.


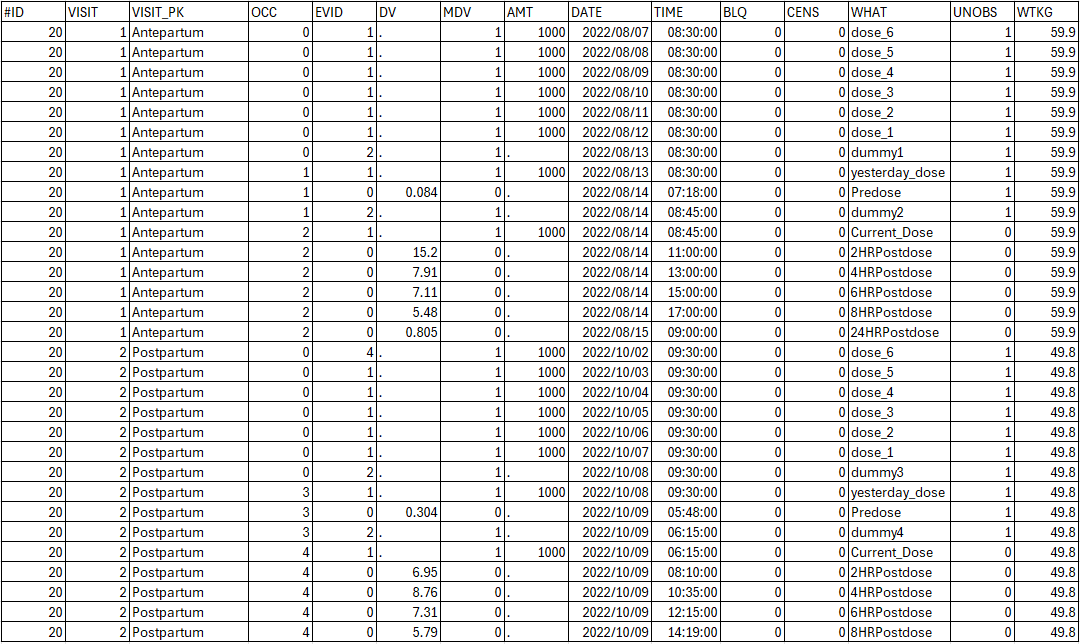


**Figure S7:** A snapshot of the NONMEM dataset for one anonymised pregnant participant showing the unobserved column (UNOBS) coded as 0 and 1, where 1 indicates that the dose was unobserved and 0 otherwise. OCC, occasion; EVID, event ID; DV, dependent variable; MDV, missing DV, 1 indicates the drug concentration is missing and 0 otherwise; AMT, amount of levofloxacin in mg; BLQ, below the limit of quantification; CENS, censored; WTKG, weight in kilograms.

**Implementation of between occasion variability and between subject variability.**

Random effects variability was quantified assuming a log normal distribution, where between subject variability (BSV) was tested on disposition parameters and between-occasion variability (BOV) on absorption parameters and bioavailability. BOV was included in the absorption parameters and bioavailability since absorption is often more variable within a patient due to factors that can differ between occasions and visits including stomach pH, gastric emptying, intestinal motility, the presence and absence of food as well as the differences in food quantity and composition, all of which can influence the absorption process. An occasion was defined as a dosing event and its associated PK samples, while a visit referred to an event where PK sampling was conducted. For example, occasion 1 is linked to the dose taken on the day prior to sampling and its associated concentration (trough sample). Occasion 2 is linked to the dose administered at the clinic on the day of sampling and the subsequent PK profile. Non-pregnant participants had only one visit with PK sampling, hence two occasions (occasion 1 and occasion 2). Pregnant participants had two visits, with two occasions per visit, hence resulting in four occasions (occasions 1 and 2 for the antepartum visit, and occasions 3 and 4 for the postpartum visit). Furthermore, while BOV accounts for variability between occasions, it also inherently captures part of the BSV within each occasion, as BSV reflects differences between participants that persist across occasions. Further illustrations on handling BOV and BSV can be found in our Population Approach Group Europe (PAGE) 2024 tutorial on this link:

<https://www.page-meeting.org/default.asp?abstract=11282>

**Dose timing and imputation of unobserved doses**

During the pharmacokinetic visits, investigators recorded the times at which participants reported taking their doses, specifically noting at least the last three doses prior to the visit. We included seven records of the unobserved doses preceding the pharmacokinetic visit in the NONMEM data set, to ensure that the data reflected that the patient was at steady state. For instances where exact dosing times were not reported (i.e., unobserved doses), we imputed the timing based on the most recent reported dosing interval for each participant, assuming that they took the drug around the same time each day. Figure S7 is a snapshot of the NONMEM dataset for one anonymised pregnant participant with two visits (antepartum and postpartum) illustrating the implementation of unobserved doses. This approach allowed us to maintain consistency in dosing intervals and account for potential variability in adherence. Recognizing the higher uncertainty associated with unobserved doses, we assessed additional variability in absorption parameters and bioavailability for these doses. The inclusion of this parameter accounted for the increased uncertainty in dose timing and dosing procedures prior to the observed dose. Implementation of this parameter on bioavailability (was more significant than on absorption parameters) is illustrated in the NONMEM code as THETA(14).

**Allometric scaling of disposition parameters**

Allometric scaling of clearance (with a fixed exponent of 0.75) and volume of distribution (with a fixed exponent of 1) parameters scaled by either weight or fat-free mass (FFM) was investigated to adjust for differences in body size^10^. The FFM formula was calculated as follows:

$$FFM= \frac{{WHS}_{max}\cdot{HT}^{2}\cdot TBW}{{WHS}_{50}\cdot{HT}^{2}+TBW}$$

Where Weight-scaled Height Maximum (WHSmax) = 37.99 for females and 42.92 for males, while Weight-scaled Height at 50% (WHS_50_) = 35.98 for females and 30.93 for males. HT is height in metres while TBW is total body weight in kilograms.

**NONMEM model code for levofloxacin**

; Settings for the memory of NONMEM

$SIZES PD=-1000 LVR=-150 LTH=-200 MAXFCN=10000000 LNP4=-150000

;--------------------------------------------------------------------------------------------------------

$PROBLEM LEVOFLOXACIN_POOLED_ANALYSIS

;--------------------------------------------------------------------------------------------------------

$INPUT ID VISIT OCC EVID DV MDV AMT DAT2=DROP TIME BLQ CENS

VPCTIME UNOBS FLAG PROBLEM PATIENT_DIFF DOSE HTM WTKG

AGEYRS SEX1M RACE1B STUDY_BEAT HIV_STATUS PREGNANT CREATININE ALBUMIN

;----------------------------------------------------------------------------------------------------------

$DATA LVX_PK_DAT.csv IGNORE=#

;----------------------------------------------------------------------------------------------------------

$ABBREVIATED DERIV2=NO ; Prevents the computation of second derivatives, which are needed only for the Laplacian method.

;---------------------------------------------------------------------------------------------------------------

$SUBROUTINE ADVAN14 TRANS1 TOL=9 ATOL=6

;---------------------------------------------------------------------------------------------------------------------

$MODEL NCOMPARTMENTS=2

COMP=(ABS DEFDOSE) ; 1 ABSORPTION COMPARTMENT

COMP=(CENTRAL DEFOBSERVATION) ; CENTRAL COMPARTMENT

;-----------------------------------------------------------------------------------------------------------------------

$PK

BOVCL = 0

BOVBIO = 0

BOVKA = 0

BOVMTT = 0

; ------- BSV

BSVCL = ETA(1)

BSVV = ETA(2)

BSVKA = ETA(3)

BSVBIO = ETA(4)

BSVV3 = ETA(5)

BSVQ = ETA(6)

BSVV4 = ETA(7)

BSVQ2 = ETA(8)

; ---------- BOV

IF(OCC==1) THEN

BOVCL = ETA(9)

BOVBIO = ETA(13)

BOVKA = ETA(17)

BOVMTT = ETA(21)

ENDIF

IF (OCC==2) THEN

BOVCL = ETA(10)

BOVBIO = ETA(14)

BOVKA = ETA(18)

BOVMTT = ETA(22)

ENDIF

IF (OCC==3) THEN

BOVCL = ETA(11)

BOVBIO = ETA(15)

BOVKA = ETA(19)

BOVMTT = ETA(23)

ENDIF

IF (OCC==4) THEN

BOVCL = ETA(12)

BOVBIO = ETA(16)

BOVKA = ETA(20)

BOVMTT = ETA(24)

ENDIF

; --------------ALLOMETRIC SCALING

; These formulas require WT in KG and HT in m !!!

AGE = AGEYRS

HT = HTM

WT = WTKG

SEX = SEX1M

; Female has value SEX=0 and male value SEX=1

IF (SEX.EQ.0) THEN ; female

WHSMAX=37.99

WHS50=35.98

ELSE ;males

WHSMAX=42.92

WHS50=30.93

ENDIF

HTM2 = HTM**2

FFM = (WHSMAX*HTM2*WT)/(WHS50*HTM2+WT)

FAT = WT-FFM

IF (FAT.LT.0) FAT = 0

; ------- Typical values of covariates

TVWT = 58 ; median weight from data set

TVFAT = 18.6

TVFFM = 39.4

;--------- Allometric scaling and covariates

;ALLMCL_WT = (WT/TVWT)**0.75

;ALLMV_WT = (WT/TVWT)

;---------------------------------------------------------------------------

;ALLMCL_FAT = (FAT/TVFAT)**0.75

;ALLMV_FAT = (FAT/TVFAT)

;-----------------------------------------------------------------------------

ALLMCL_FFM = (FFM/TVFFM)**0.75

ALLMV_FFM = (FFM/TVFFM)

;----------------------------------------------------------------------------------

;------------- Effect of serum creatinine on clearance----------------

MEDCREAT = 56.2 ; Median serum creatinine from the data set

CR_CL = (CREATININE /MEDCREAT)**THETA(15)

;---------------Scaling BOVBIO on unobserved doses--------------

E_BOVF = THETA(14)

IF(UNOBS.EQ.1) THEN

BOVBIO = E_BOVF*BOVBIO

ENDIF

;------------Effect of pregnancy on clearance------------

preg_CL = 1

IF (PREGNANT.EQ.1) preg_CL = 1 + THETA(12)

;---------Typical values---------------------------------------------------------------------------------------------

TVCL = THETA(1)*ALLMCL_FFM*preg_CL*CR_CL

TVV = THETA(2)*ALLMV_FFM

TVKA = THETA(3)

TVBIO = THETA(4)

TVMTT = THETA(7)

TVV3 = THETA(8)

TVQ = THETA(9)

TVV4 = THETA(10)

TVQ2 = THETA(11)

TVNN = THETA(13)

;-----------Define parameters-------------------------------------------------------------------------------------

CL = TVCL*EXP(BSVCL+BOVCL) ; CLEARANCE

V = TVV*EXP(BSVV) ; CENTRAL VOL.

KA = TVKA*EXP(BSVKA+BOVKA) ; ABS. RATE CONSTANT

BIO = TVBIO*EXP(BSVBIO+BOVBIO) ; BIOAVAILABILITY

MTT =TVMTT*EXP(BOVMTT) ; TRANSIT TIME

V3 = TVV3*EXP(BSVV3) ; PERIPH VOL1

Q = TVQ*EXP(BSVQ) ; INTER COMPT CL1

V4 = TVV4*EXP(BSVV4) ; PERIPH VOL2

Q2 = TVQ2*EXP(BSVQ2) ; INTER COMPT CL2

NN = TVNN ; Number of transit compartments

;-----------------------------------------------------------------------------------------------------------------------

; re-parameterization

K = CL/V ;(rate constant of elimination)

K23 = Q/V ; (rate constant from central to peripheral 1)

K32 = Q/V3 ;(rate constant from peripheral 1 to central)

K24 = Q2/V ;(rate constant from central to peripheral 2)

K42 = Q2/V4 ; (rate constant from peripheral 2 to central)

;----------------------------------------------------------------------------------------------------------------------

; Transit compartment absorption. Refer to : “Savic RM, Jonker DM, Kerbusch T, Karlsson MO. Implementation of a transit compartment model for describing drug absorption in pharmacokinetic studies. J Pharmacokinet Pharmacodyn. 2007 Oct;34(5):711-26. doi: 10.1007/s10928-007-9066-0. Epub 2007 Jul 26. PMID: 17653836.”

F1 = 0 ;

KTR = (NN+1)/MTT

IF (NEWIND/=2.OR.EVID>=3) THEN

TNXD = TIME

PNXD = AMT

ENDIF

TDOS = TNXD

PD = PNXD

IF(AMT>0) THEN

TNXD=TIME

PNXD=AMT

ENDIF

; To speed up the computation, I calculate here all the non-time-varying quantities used in $DES

PIZZA = LOG(BIO*PD*KTR + 1E-12) - GAMLN(NN+1) ; without +0.00001, it won't work with ETAs in bioavailability

A_0(1) = 1E-12

A_0(2) = 1E-12

;-----------------------------------------------------------------------------

$DES

TEMPO = T-TDOS ; this is time after dose for the transit, it should always be >= 0

KTT = 0

TRANSIT = 0

IF(PD.GT.0.AND.TEMPO.GT.0) THEN ; This happens only if PD>0, so only if a dose has been detected

KTT = KTR*(TEMPO)

TRANSIT = EXP(PIZZA+NN*LOG(KTT)-KTT)

ENDIF

DADT(1) = TRANSIT -KA*A(1)

DADT(2) = KA*A(1) -K*A(2)

;-------------------------------------------------------------------------------------------------------------------------------------

$ERROR

IPRED=A(2)/V

LLOQ = 0.0781 ; DEFINE YOUR OWN LLOQ HERE

PROP = IPRED*THETA(5)

ADD = THETA(6) + (LLOQ*0.2) ; The lower bound of THETA(.) can be zero, if it goes there, we can fix it to zero and ADD will be 20% of LLOQ

; For CENS==1 (i.e. first CENSORED value in a series, which was imputed to LLOQ/2), we add extra additive error on the concentrations,

; since the value in DV has been imputed and therefore more uncertain.

IF (ICALL/=4.AND.CENS==1) THEN

ADD = ADD +(LLOQ*0.5)

ENDIF

NO_FIT = 0

; For CENS==2 (i.e. the trailing CENSORED values in a series that were imputed to LLOQ/2), ;we don't want these to influence the fit, we only want them for simulation-based diagnostics ;such as the VPC. So we define a separate error structure for these points. It has no proportional ;component (PROP = 0, as we would not want these points to affect our estimate of proportional ;error) and a FIXED and HUGE additive component (ADD = 1000000000, large with respect ;to the readings of concentration)

IF (ICALL/=4.AND.CENS==2) THEN; discard consecutive BLQ during estimation

PROP = 0

ADD = 10000000000

NO_FIT = 1

ENDIF

W = SQRT(ADD**2+PROP**2); using add + prop error model

; Protective code

IF (W.LE.0.000001) W=0.000001

IRES=DV-IPRED

IWRES=IRES/W

Y = IPRED + W*ERR(1)

IF (ICALL==4.AND.Y<=LLOQ) Y = LLOQ/2; To prevent simulation (ICALL==4) of negative values.

;-------------------------------------------------------------------------------------------

IF(AMT>0) THEN

TIMEDOSE = TIME

AMOUNTDOSE = AMT

ENDIF

TAD = TIME-TIMEDOSE; To calculate time after dose.

AUC_INF = (DOSE*BIO)/CL; Calculating AUC

;---------------------------------------------------------------------------------

VARCL = BSVCL + BOVCL

VARBIO = BSVBIO + BOVBIO

VARAUC = BSVBIO + BOVBIO - BSVCL - BOVCL

VARABS = BOVKA + BSVKA - BOVMTT

;------------------------------------------RETRIEVE AMOUNT IN EACH COMPARTMENT--------------------

AA1 = A(1)

AA2 = A(2)

;-----------------------------------------------------------------------------------------------------------------------

$THETA (0,6.05859,90) ; 1 CL [L/h]

$THETA (0,85.8803,800) ; 2 V [L]

$THETA (0,1.59498,5) ; 3 KA [1/h]

$THETA 1 FIX ; 4 BIO

$THETA (0,0.0733236,0.5) ; 5 PROP []

$THETA (0,0.243823,0.5) ; 6 ADD [mg/L]

$THETA (0,1.07437,3) ; 7 MTT

$THETA (0,0,800) FIX ; 8 V3 [L]

$THETA (0,0,90) FIX ; 9 Q [L/h]

$THETA (0,0,800) FIX ; 10 V4 [L]

$THETA (0,0,90) FIX ; 11 Q2 [L/h]

$THETA (0,0.380866) ; 12 preg_CL

$THETA (0.01,20) FIX ; 13 NN []

$THETA (0,2.35136,5) ; 14 E_BOVF

$THETA (-0.5,-0.366504,0.0333) ; 15 CR_CL

;-----------------------------------------------------------------------------------------------------------------------

$OMEGA BLOCK(1)

0.0489868 ; 1 BSV CL

$OMEGA BLOCK(1) FIX

0 ; 2 BSV V

$OMEGA BLOCK(1) FIX

0 ; 3 BSV KA

$OMEGA BLOCK(1) FIX

0 ; 4 BSV BIO

$OMEGA BLOCK(1) FIX

0 ; 5 BSVV3

$OMEGA BLOCK(1) FIX

0 ; 6 BSVQ

$OMEGA BLOCK(1) FIX

0 ; 7 BSVV4

$OMEGA BLOCK(1) FIX

0 ; 8 BSVQ2

;-----------------------------------------------------------------------------------------------------------------------

$OMEGA BLOCK(1) FIX

0 ; 9 BOVCL

$OMEGA BLOCK(1) SAME

$OMEGA BLOCK(1) SAME

$OMEGA BLOCK(1) SAME

;-----------------------------------------------------------------------------------------------------------------------

$OMEGA BLOCK(1)

0.0562092 ; 13 BOVBIO

$OMEGA BLOCK(1) SAME

$OMEGA BLOCK(1) SAME

$OMEGA BLOCK(1) SAME

;-----------------------------------------------------------------------------------------------------------------------

$OMEGA BLOCK(1)

0.732947 ; 17 BOVKA

$OMEGA BLOCK(1) SAME

$OMEGA BLOCK(1) SAME

$OMEGA BLOCK(1) SAME

;-----------------------------------------------------------------------------------------------------------------------

$OMEGA BLOCK(1)

0.211291 ; 21 BOVMTT

$OMEGA BLOCK(1) SAME

$OMEGA BLOCK(1) SAME

$OMEGA BLOCK(1) SAME

;-----------------------------------------------------------------------------------------------------------------------

$SIGMA 1 FIX

$ESTIMATION MSFO=run520.msf MAXEVAL=9999 PRINT=1 METHOD=1 INTER

NOABORT NSIG=3 SIGL=9 NONINFETA=1 ETASTYPE=1 ; REPEAT

$COVARIANCE PRINT=E MATRIX=S

$TABLE ;-----------------------------------------------------------------------------------------------

**REFERENCES**

1. Van’t Boveneind-Vrubleuskaya Natasha, Seuruk Tatiana HK Van. Pharmacokinetics of Levofloxacin in Multidrug- and Extensively Drug-Resistant Tuberculosis Patients. *Antimicrob Agents Chemother*. 2017;61(8):1-10.

2. Peloquin CA, Phillips PPJ, Mitnick CD, et al. Increased doses lead to higher drug exposures of levofloxacin for treatment of tuberculosis. *Antimicrob Agents Chemother*. 2018;62(10):1-5. doi:10.1128/AAC.00770-18

3. Mohamed S, Mvungi HC, Sariko M, et al. Levofloxacin pharmacokinetics in saliva as measured by a mobile microvolume UV spectrophotometer among people treated for rifampicin-resistant TB in Tanzania. *J Antimicrob Chemother*. 2021;76(6):1547-1552. doi:10.1093/jac/dkab057

4. Sidamo T, Rao PS, Aklillu E, et al. Population Pharmacokinetics of Levofloxacin and Moxifloxacin, and the Probability of Target Attainment in Ethiopian Patients with Multidrug-Resistant Tuberculosis. *Infect Drug Resist*. 2022;15:6839-6852. doi:10.2147/IDR.S389442

5. Van den Elsen SHJ, Sturkenboom MGG, Van’t Boveneind-Vrubleuskaya N, et al. Population pharmacokinetic model and limited sampling strategies for personalized dosing of levofloxacin in tuberculosis patients. *Antimicrob Agents Chemother*. 2018;62(12). doi:10.1128/AAC.01092-18

6. Tsuruoka S, Yokota N, Hayasaka T, Saito T, Yamagata K. Pharmacokinetics of multiple-dose levofloxacin in hemodialysis patients. *Am J Kidney Dis*. 2011;58(3):498-499. doi:10.1053/j.ajkd.2011.05.014

7. Deshpande D, Pasipanodya JG, Mpagama SG, et al. Levofloxacin Pharmacokinetics/Pharmacodynamics, Dosing, Susceptibility Breakpoints, and Artificial Intelligence in the Treatment of Multidrug-resistant Tuberculosis. *Clin Infect Dis*. 2018;67(Suppl 3):S293-S302. doi:10.1093/cid/ciy611

8. Yu X, Wang G, Chen S, et al. Wild-Type and Non-Wild-Type Mycobacterium tuberculosis MIC Distributions for the Novel Fluoroquinolone Antofloxacin Compared with Those for Ofloxacin, Levofloxacin, and Moxifloxacin. 2016;60(9):5232-5237. doi:10.1128/AAC.00393-16.Address

9. Fish DN, Chow AT. The Clinical Pharmacokinetics of Levofloxacin. *Clin Pharmacokinet*. 1997;32(2):101-119. doi:10.2165/00003088-199732020-00002

10. Anderson BJ, Holford NHG. Mechanism-based concepts of size and maturity in pharmacokinetics. *Annu Rev Pharmacol Toxicol*. 2008;48:303-332. doi:10.1146/annurev.pharmtox.48.113006.094708
